# Menopausal hormone therapy and other breast cancer risk factors in relation to the risk of different histological subtypes of breast cancer: a case-control study

**DOI:** 10.1186/bcr1378

**Published:** 2006-02-17

**Authors:** Lena U Rosenberg, Cecilia Magnusson, Emma Lindström, Sara Wedrén, Per Hall, Paul W Dickman

**Affiliations:** 1 Department of Medical Epidemiology and Biostatistics, Karolinska Institutet, S-171 77 Stockholm, Sweden; 2 Department of Public Health Sciences, Karolinska Institutet, Norrbacka, S-171 76 Stockholm, Sweden; 3 Population Genetics, Genome Institute of Singapore, Biopolis Street, #02-01 Genome, S138672, Singapore

## Abstract

**Introduction:**

Breast cancers of different histology have different clinical and prognostic features. There are also indications of differences in aetiology. We therefore evaluated the risk of the three most common histological subtypes in relation to menopausal hormone therapy and other breast cancer risk factors.

**Methods:**

We used a population-based case-control study of breast cancer to evaluate menopausal hormone therapy and other breast cancer risk factors for risk by histological subtype. Women aged 50 to 74 years, diagnosed with invasive ductal (n = 1,888), lobular (n = 308) or tubular (n = 93) breast cancer in Sweden in 1993 to 1995 were compared with 3,065 age-frequency matched controls randomly selected from the population. Unconditional logistic regression was used to calculate odds ratios (ORs) and 95% confidence intervals (CIs) for ductal, lobular, and tubular cancer.

**Results:**

Women who had used medium potency estrogen alone were at increased risks of both ductal and lobular cancer. Medium potency estrogen-progestin was associated with increased risks for all subtypes, but the estimates for lobular and tubular cancer were higher compared with ductal cancer. We found OR 5.6 (95% CI 3.2–9.7) for lobular cancer, OR 6.5 (95% CI 2.8–14.9) for tubular cancer and OR 2.3 (95% CI 1.6–3.3) for ductal cancer with ≥5 years use of medium potency estrogen-progestin therapy. Low potency oral estrogen (mainly estriol) appeared to be associated with an increased risk for lobular cancer, but the association was strongest for short-term use. Reproductive and anthropometric factors, smoking, and past use of oral contraceptives were mostly similarly related to the risks of the three breast cancer subtypes. Recent alcohol consumption of > 10 g alcohol/day was associated with increased risk only for tubular cancer (OR 3.1, 95% CI 1.4–6.8).

**Conclusion:**

Menopausal hormone therapy was associated with increased risks for breast cancer of both ductal and lobular subtype, and medium potency estrogen-progestin therapy was more strongly associated with lobular compared with ductal cancer. We also found medium potency estrogen-progestin therapy and alcohol to be strongly associated with tubular cancer. With some exceptions, most other risk factors seemed to be similarly associated with the three subtypes of breast cancer.

## Introduction

Use of menopausal hormone therapy has been shown to increase the risk of breast cancer[1], and data indicate that combined medium potency estrogen-progestin therapy (mainly estradiol or conjugated estrogens combined with progestin) is associated with a higher risk for breast cancer than medium potency estrogen alone therapy [2,3]. The incidence rate of lobular breast cancer has increased more rapidly than that of ductal breast cancer during the past 30 years. [4,5], coinciding with the introduction and rising use of menopausal hormone therapy. Furthermore, studies from the United States have rather consistently found medium potency estrogen-progestin therapy to be more strongly associated with lobular than with ductal breast cancer risk [6-11]. It is not clear, however, whether use of menopausal hormone therapy also increases the risk of ductal breast cancer, or whether medium potency estrogen alone therapy has differential impacts on lobular and ductal breast cancer risk. Apart from one study reporting a strong association with menopausal hormone therapy [12], little is known about the aetiology of tubular breast cancer. Whether low potency oral estrogen (oral estriol without progestin) or local estrogen (cream or pessary, without progestin) are associated with certain histological subtypes of breast cancer has, to our knowledge, not been studied before. Lastly, the influence of other breast cancer risk factors on histological subtypes of breast cancer is not well known [13-17].

We report results on these relationships from a large Swedish case-control study. The types of estrogens and gestagens used for menopausal hormone therapy differ between countries and, to our knowledge, this is the first study outside the United States to report in detail on these associations.

## Materials and methods

### Subjects

This study is an extension of a case-control study among all Swedish residents born in Sweden and aged 50 to 74 years between 1 October 1993 and 31 March 1995 [18-21]. The study was approved by the ethical review board at the Karolinska Institute, and by the five ethical review boards in other regions in Sweden. Women with incident primary invasive breast cancer were identified via the six Swedish Regional Cancer Registries. The women were contacted via their doctors and asked for written consent to be approached with a mailed questionnaire. The study identified 3,979 women with a diagnosis of invasive breast cancer, of whom 84% (3,345) participated. The primary reasons for non-participation were patient's refusal or doctor's refusal because of the patient's poor health. The mean interval from diagnosis to data collection was 4.3 months (standard deviation 1.5 months).

Controls were frequency matched by the expected age distribution among cases and identified through the Swedish National Population Register holding data on national registration number, name, address, and place of birth of all Swedish residents. The response rate among controls was 82% (3,455/4,188). Women previously diagnosed with invasive cancer (other than non-melanoma skin cancer) were excluded from the study (112 cases and 91 controls). Menopause was defined as the age at last menstrual period or age at bilateral oophorectomy, if one year or more prior to data collection. Premenopausal women (198 cases and 152 controls), women below the age of 55 years with unknown age at menopause considered premenopausal (202 cases and 101 controls), and women with missing information on body mass index (BMI; 14 cases and 45 controls) or age at first birth (5 cases and 1 control) were excluded.

In a second phase of the study, we retrieved information about histology and various other tumour characteristics from the medical records of all participating cases. Following a decision of the ethical review board of the University of Lund, written informed consent to retrieve this information was sought from cases in that region (n = 563), among whom 58 women did not provide informed consent. The medical records for 31 study participants could not be found.

Information from the medical records led us to exclude a further 58 cases with non-invasive breast cancer, 35 cases with previous cancer, one case with a cancer diagnosis other than breast cancer, and 19 cases diagnosed before or after the study period. The final study comprised 2,643 breast cancer cases and 3,065 controls.

### Data collection

Data on sociodemographic, anthropometric, reproductive and menstrual factors, use of oral contraceptives, medical history, lifetime physical activity and smoking habits, as well as recent (one year before data collection) dietary habits and alcohol use were collected by means of a postal questionnaire. Detailed information on use of menopausal hormone therapy, including timing and type of hormones for each treatment episode, was also requested and a colour chart displaying all preparations ever marketed in Sweden was included with the questionnaire to aid recall. In addition, approximately 50% of both cases and controls were contacted by telephone to complete missing or ambiguous responses, mainly on use of menopausal hormone therapy. Of all eligible controls, 14% did not return the questionnaire, but agreed to a telephone interview covering the most important items, including use of menopausal hormone therapy.

We obtained information on tumour histology from the pathology report in the medical record. Histology was classified after the invasive component as ductal (n = 1,888), lobular (n = 308), tubular (≥90% tubular component, n = 93), mixed lobulo-ductal (n = 58), medullary (n = 29), mucinous (n = 61), papillary (n = 10), adenocarcinoma not otherwise specified (n = 122), or unspecified tumours (n = 38). Information on histology was missing in 36 cases because the medical record was not identified, or the woman had not had a breast cancer operation. We analysed ductal, lobular, and tubular cases and all other cases were excluded from the analyses. Adenocarcinoma not otherwise specified was not analysed as we considered it an undefined subtype, and the other histology subtypes were too few to be analysed separately.

### Statistical analyses

We used unconditional logistic regression to estimate odds ratios (ORs) with associated 95% confidence intervals (95% CIs) separately for ductal, lobular, and tubular cancer cases compared with controls. To formally test whether effect estimates were different for lobular or tubular cancer compared with ductal cancer we fitted models comparing lobular or tubular cases with ductal cases. For the tests comparing lobular or tubular cancer with ductal cancer, a two-sided *p*-value < 0.05 was considered a significant difference.

Use of menopausal hormone therapy was categorized according to type as medium potency estrogen alone (mainly estradiol or conjugated estrogens), medium potency estrogen with progestin, progestin alone (without concomitant estrogen), low potency oral estrogen (oral estriol without progestin), or local estrogen (cream or pessary, without progestin) for each treatment episode. Medium potency estrogen-progestin therapy was also classified according to regimen, that is, as sequential (progestin taken less than 16 days per 28 days) or continuous use (19 or more days per 28 days). We censored all exposure after a reference date, defined in cases as date of diagnosis minus 3 months, and in controls as the date of questionnaire arrival minus mean time from diagnosis to questionnaire arrival in cases, minus an additional 3 months.

We studied the effect of ever use, duration and recency of use. Analyses of the associations between use of specific types of menopausal hormone therapy and the risk of breast cancer were performed both for non-exclusive use (all users of that specific therapy), and restricted to exclusive users of that particular type, with never users of any menopausal hormone therapy as the reference group. No more than five women reported exclusive current use of progestin alone therapy, so we were unable to evaluate this therapy. Only 31 women had simultaneously used progestin with low potency oral estrogen or local estrogen, so this combination was not analysed separately, but these women were excluded from all analyses on exclusive use.

In the analyses on menopausal hormone therapy, women with an unknown age at menopause were excluded (296 cases and 301 controls) to avoid bias leading to underestimation of the associations as first pointed out by Pike and colleagues [22].

We adjusted all estimates for age. We also assessed the potential confounding effects of a range of other variables, including number of births, age at first birth, age at menopause, surgical menopause (bilateral oophorectomy), menopausal symptoms, BMI (weight(kg)/height (m)^2^) one year before data collection, height, socioeconomic status, smoking, and ever use of medium potency estrogen-progestin therapy. Confounding was defined as changing the point estimates more than 10% from the age-adjusted values. Only covariates confounding the effect of at least one of the exposures in the same table were included in the models. In addition, age at first birth and parity were adjusted for each other.

The analyses of the influence of age at menopause on the risk of different breast cancer subtypes were restricted to women with a known age at menopause that had not used menopausal hormone therapy apart from local estrogen before menopause in order to reduce confounding. The influence of BMI one year before data collection and the risks of different breast cancer subtypes was evaluated only among never users of menopausal hormone therapy apart from local estrogen – as BMI and menopausal hormone therapy are known to interact biologically to cause breast cancer [20]. This interaction was studied in another approach, where study participants were cross-classified by their use of medium potency estrogen-progestin therapy and BMI, and the risks of ductal and lobular cancer were calculated. Analyses were performed using the SAS System, version 9.1 (SAS Institute, Carey, NC, USA).

## Results

Controls were slightly older than cases (Table 1). Surgical menopause was equally common among cases and controls. Menopausal symptoms were slightly more common among cases than controls. Cases, especially tubular cancer cases, were more frequently of high socioeconomic status than controls. Ever use of menopausal hormone therapy (all types combined) was more common among cases than controls. Among users of medium potency estrogen, estradiol (mainly 2 mg orally/day or 50 μg transdermally/day) was most frequently used and per oral administration (tablets) was most common. When progestin was used, testosterone-derived progestins were most common (mainly 1 mg norethisterone acetate/day if added continuously; and either 250 μg levonorgestrel/day or 1 mg norethisterone acetate/day in 10 of 28 days if added cyclically). Use of low potency oral estrogen therapy constituted almost principally 1 mg estriol per day and was more common among lobular cases than other cases and controls, and local estrogen therapy consisted of cream or pessary containing estradiol or estriol. Local estrogen therapy was equally common among cases and controls.

### Medium potency estrogen alone

Use of medium potency estrogen alone was similarly associated with increased risks of ductal and lobular cancer (Table 2). Due to few cases, tubular cancer was not possible to evaluate. Exclusive ever use of medium potency estrogen alone was similarly associated with the risks of ductal (OR 2.0, 95% CI 1.5–2.9) and lobular (OR 2.4, 95% CI 1.3–4.6) breast cancer. Use for more than five years entailed ORs of 2.9 (95% CI 1.6–5.2) and 3.1 (95% CI 1.0–9.5) for ductal and lobular breast cancer, respectively (Table 2). Ductal and lobular cancers were both associated with current use, and ductal cancer in addition with past use, but without significant differences between ductal and lobular cancer.

### Medium potency estrogen plus progestin

Use of medium potency estrogen-progestin was associated with increased risks of all three subtypes of breast cancer, with the highest risks among ≥5 years and current exclusive use (Table 2). These risks were significantly higher among lobular and tubular cancer compared with ductal cancer. Women who used medium potency estrogen-progestin for ≥5 years (of whom > 80% were current users) had an OR of 2.3 for ductal cancer (95% CI 1.6–3.3), OR 5.6 for lobular cancer (95% CI 3.2–9.7), and OR 6.5 (95% CI 2.8–14.9) for tubular cancer compared with those who never used menopausal hormone therapy (Table 2). Among past users, no clear association with any cancer subtype was discerned.

### Sequential and continuous medium potency estrogen-progestin

Non-exclusive users of both sequential and continuous medium potency estrogen-progestin had a median duration treatment of four years, compared with two years for exclusive users (data not shown). This was reflected in the higher point estimates for non-exclusive compared with exclusive ever use for lobular and tubular breast cancer (Table 2). Exclusive use of sequential medium potency estrogen-progestin therapy was not significantly differently associated with ductal, lobular or tubular cancer. Exclusive use of continuous medium potency estrogen-progestin therapy, on the other hand, was significantly more strongly associated with lobular compared with ductal cancer, when measured as ever use, <5 years use, and current use. Continuous use ≥5 years was associated with high point estimates for lobular (OR 5.9, 95% CI 2.5–14.0) and tubular (OR 8.2, 95% CI 2.4–27.5) cancer, but not significantly different from ductal cancer (OR 2.7, 85% CI 1.5–4.7). For lobular cancer the different risk estimates for exclusive ever use of continuous versus sequential use did not reach statistical significance (*p* = 0.36).

### Low potency estrogen

Non-exclusive and exclusive ever use of low potency oral estrogen was associated with an increased risk for lobular (OR 2.0, 95% CI 1.3–3.2) but not ductal (OR 1.2, 95% CI 0.9–1.5) or tubular (OR 1.0, 95% CI 0.3–2.8) breast cancer (Table 2). The increased risk was confined to <5 years of use and past users.

### Local estrogen

We found no associations between non-exclusive or exclusive use of local low potency estrogen therapy and either ductal or lobular breast cancer (Table 2). The estimates for tubular cancer were non-significantly above unity, with no trend of increased estimates for longer duration.

### Reproductive factors

The risks associated with parity, age at menarche and age at menopause did not vary significantly between the three histological subtypes (Table 3). Increasing number of births was associated with a decreased risk of ductal but not of lobular cancer. Age at first birth was slightly more associated with lobular than with ductal cancer, but the difference was not significant.

### Anthropometric factors

Height was similarly associated with the three subtypes. Recent BMI and adult weight gain, evaluated among never users of menopausal hormone therapy apart from local estrogen, were associated with similar ORs for ductal and lobular cancer (Table 3). The association between recent BMI and tubular cancer was close to unity, but not statistically different from the estimates for ductal cancer.

### Other factors

Having a mother or sister with breast cancer seemed more strongly associated with lobular (OR 2.4, 95% CI 1.8–3.4) than with ductal (OR 1.8, 95% CI 1.5–2.2) breast cancer (*p* for heterogeneity = 0.06). Previous operation due to benign breast disease was associated with slightly higher risk for lobular (OR 1.9, 95% CI 1.4–2.6) compared with ductal (OR 1.5, 95% CI 1.2–1.8) cancer. The OR for tubular cancer was near unity, but not statistically different from ductal cancer. Use of oral contraceptives (99% of all users stopped v5 years before the reference date) was not associated with any of the three histological subtypes of breast cancer among these postmenopausal women (Table 3). Recent high alcohol consumption was associated with tubular breast cancer (OR for ≥10 g/day 3.1, 95% CI 1.4–6.8). For lobular cancer a tendency of higher ORs with increasing alcohol consumption was seen, but no category had a significantly increased OR (Table 3). We found no indication of an association between alcohol consumption and ductal cancer. Recent smoking was borderline associated with risk of ductal cancer (OR 1.2, 95% CI 1.0–1.4), and the OR for tubular cancer was significantly lower compared with ductal cancer (OR 0.8, 95% CI 0.5–1.2) but not significantly different compared with controls (Table 3).

### Interaction between medium potency estrogen-progestin use and BMI

We stratified exclusive ever use of medium potency estrogen-progestin into three categories of BMI (Table 4). The increased risk for both ductal and lobular breast cancer with medium potency estrogen-progestin use seemed to be confined to women with BMI ≤27. Among women with BMI < 22, exclusive ever users of medium potency estrogen-progestin had OR 2.0 (95% CI 1.2–3.3) for ductal cancer and OR 3.4 (95%CI1.4–8.3) for lobular cancer compared with never users of menopausal hormone therapy

## Discussion

We found increased risks of both ductal and lobular breast cancer among women who used medium potency estrogen alone or in combination with progestin. Medium potency estrogen-progestin therapy, but not medium potency estrogen alone therapy, was significantly more strongly related to lobular than to ductal breast cancer. The risk with medium potency estrogen-progestin therapy was confined to women with a BMI ≤27. We found a stronger association with medium potency estrogen-progestin therapy for tubular cancer compared with ductal cancer. For most other risk factors, we found no strong variations in the associations with the three subtypes, but some indications of difference for parity, BMI, family history and recent alcohol consumption.

Some previous studies have found medium potency estrogen alone therapy to be associated only with lobular cancer [6,10,11], while others failed to find any association with either ductal or lobular breast cancer [8,23]. In contrast to these results, we found an increased risk for both subtypes of cancer with use of medium potency estrogen alone therapy. Schairer and colleagues [2] reported increased risk of ductal cancer with medium potency estrogen alone therapy among lean, but not obese women. BMI is known to interact biologically with estrogen therapy, thereby affecting the association of estrogen with breast cancer risk, shown for example in the Million Women Study [3]. A high average BMI is thus a possible explanation for the lack of increased breast cancer risk with conjugated estrogen alone found in the Women's Health Initiative study [24].

Our finding of a stronger association between use of medium potency estrogen-progestin therapy and the risk of lobular compared with ductal breast cancer is consistent with published results from all previous studies [6-11] except one [23]. In contrast, the increased risk also for ductal cancer after use of medium potency estrogen-progestin therapy in our data corroborates some [9,10,23], but not all [6,8,11], published studies. One possible explanation for our stronger associations between medium potency estrogen with or without progestin therapy and both ductal and lobular cancer compared with others could be differences regarding the hormones used in our study and in the United States. In our study, estradiol and higher dose testosterone-derived progestins were most commonly used, in contrast to mainly conjugated estrogens and lower dose progesterone-derived progestins in the United States [25]. Previously published results from our case control study showed that testosterone-derived progestins were more strongly related to breast cancer than progesterone-derived progestins. [20].

Two Scandinavian prospective cohort studies have analysed menopausal and subsequent risk of histological subtypes of breast cancer [12,26]. Both found higher risks for lobular compared with ductal cancer, but only Tjønneland and colleagues found an increased risk also for ductal cancer.

Newcomer and colleagues [11] reported tubular cancer to make up around 1% of diagnosed breast cancer cases in their study. They found increased point estimates for tubular cancer with estrogen alone and past use of estrogen-progestin, but the power was low. Interestingly, a Swedish cohort study [12] reported 10 tubular cases out of 131 invasive breast cancers, and use of menopausal hormone therapy (unspecified) was associated with a relative risk of 4.81 (95% CI 1.37–16.8) for tubular cancer. Gapstur and colleagues [27] grouped tumours said to be of favourable histology together (22% were tubular), and reported an association with menopausal hormone therapy. We defined histology according to the pathology report, and found 3.5% of invasive cancers to be purely tubular. Cases with mixed tubuloductal histology were classified as ductal. Our finding of a strong association between medium potency estrogen-progestin therapy and tubular cancer is interesting as menopausal hormone therapy has been associated with low grade tumours [12,28], and tubular cancers are known to have an excellent prognosis. [29]. Studies pooling data from several centres might shed further light on the risk factor patterns in this relatively rare form of breast cancer.

Three studies have reported on the impact of sequential versus continuous estrogen-progestin therapy on ductal and lobular breast cancer risk. In accordance with our results, Chen and colleagues [7] and Daling and colleagues [8] found non-significantly higher risks for lobular cancer after use of continuous than after sequential therapy whereas Li and colleagues [10] found no differences.

To our knowledge, this is the first study to report on the risk of different histological subtypes of breast cancer after use of low potency oral estrogen or local estrogen therapy. These therapies are rather common in Sweden, and the indications mainly include local vaginal symptoms and urinary tract infections. Our finding of a significantly increased risk for lobular cancer among women who used low potency oral estrogen is noteworthy, and tentatively important with regard to the biological mechanisms of breast carcinogenesis. However, this result must be interpreted cautiously since the pattern of association according to duration and recency of use is contradictory to what is seen with other types of hormone therapy. Oral estriol is quickly metabolised, so the estrogenic effect of one dose is short-term [30], but it does have systemic effects [31], and oral estriol has been associated with increased risk for endometrial cancer [32]. Locally administered estrogens also have systemic effects [30], but were not found to be associated with any of the subtypes of breast cancer in our study.

Few studies have addressed whether risk factors for breast cancer other than menopausal hormone therapy are differentially associated with the risks of ductal and lobular breast cancer. Age at first birth was non-significantly more strongly associated with lobular than ductal breast cancer in our study as well as in three [15,16,33] of four [17] previous studies. Increasing number of births seemed slightly more related to ductal compared with lobular cancer in our study, supported by two studies [16,33], whereas two studies found no differences at all [15,17].

We found no association at all between at least 5 years use of oral contraceptives and ductal, lobular or tubular cancer, but only 1% of our postmenopausal participants had last use of oral contraceptives within 5 years. Newcomer and colleagues [34] found an association with lobular cancer for users with <5 years since last use, whereas Li and colleagues [17] reported a borderline association between ≥5 years use of oral contraceptives and lobular cancer (OR 1.6, 95% CI 1.0–2.6) in women aged at least 65 years.

The tentatively stronger association between a positive family history of breast cancer and lobular compared with ductal breast cancer is supported by others [13,15,35] but not by a recent Swedish register-based study [36]. Lobular cancers are more often bilateral [37], and it could be that genetic determinants are more important in the aetiology of lobular than ductal breast cancer. Li and colleagues [38] have previously reported a significant association between alcohol consumption and lobular, but not ductal, breast cancer. We had a lower median consumption of alcohol in our study, but our point estimates give weak support to their finding. In addition, we found alcohol to be strongly associated with tubular cancer, with increasing trend over categories, a finding previously not reported in the literature.

Our study is population-based and large with high response rates (84% for cases and 82% for controls), and detailed information on use of menopasual hormone therapy and on other breast cancer risk factors. Still, for lobular and even more for tubular cancer, the number of cases is small, and chance variation may play an important role. Another possible limitation is that exposure information was self-reported and collected retrospectively. We cannot rule out the possibility that some women have reported use of medium potency estrogen alone while they actually have used medium potency estrogen-progestin. Such misclassification would possibly explain our strong associations between medium potency estrogen alone therapy and breast cancer, but the similar risks for both ductal and lobular cancer differ from what we found for medium potency estrogen-progestin therapy. Also, the concordance between self-reported use of menopausal hormone therapy and information from medical records has been shown to be satisfactorily high [39,40], and we used a colour chart of all preparations ever marketed in Sweden to aid recall. As in all retrospective studies, recall bias is of concern with regard to the validity of our results. Reassuringly, use of sequential and continuous combined medium potency estrogen-progestin were demonstrated to have opposite impacts on the risk of endometrial cancer in a parallel study using the same set of controls [41]. Regarding the case/case comparisons, recall differences due to different histological type is unlikely.

Another limitation is that several pathologists at different laboratories did the histological classifications, which may have resulted in non-differential misclassification, possibly diluting the associations.

Many risks are assessed in this study, but we believe it is more relevant to discuss the results in relation to previous results and biological credibility than to adjust for multiple comparisons in the statistical analyses.

## Conclusion

Most risk factors seem to affect ductal, lobular and tubular breast cancer similarly, but there is accumulating evidence for medium potency estrogen-progestin therapy as a stronger risk factor for lobular than ductal breast cancer. We also found significant associations between medium potency estrogen alone therapy and both ductal and lobular cancer, and between medium potency estrogen-progestin therapy and ductal breast cancer. Our findings of an association between low potency oral estrogen and lobular breast cancer, as well as the strong associations between tubular cancer and both medium potency estrogen-progestin therapy and alcohol intake is notable but has yet to be confirmed or refuted by other studies.

## Abbreviations

BMI = body mass index; CI = confidence interval; OR = odds ratio.

## Competing interests

The authors declare that they have no competing interests.

## Authors' contributions

CM and PH identified the initial hypothesis. LR, CM and PH were involved in data collection. LR, EL and PD performed the statistical analyses. LR, CM, SW, PH and PD interpreted the data. LR and EL drafted the manuscript, and CM, SW, PH and PD revised the manuscript. All authors read and approved the final version of the manuscript.

## Figures and Tables

**Table 1:** Selected characteristics of the study population

Characteristic	Controls (n = 3,065)	Ductal cancer (n = 1,888)	Lobular cancer (n = 308)	Tubular cancer (n = 93)
	No. (%)	No. (%)	No. (%)	No. (%)
Age (years)				
50–54	233 (8)	195 (10)	31 (10)	9 (10)
55–59	582 (19)	431 (23)	70 (23)	20 (22)
60–64	678 (22)	390 (21)	61 (20)	26 (28)
65–69	790 (26)	473 (25)	73 (24)	23 (25)
70–74	782 (26)	399 (21)	73 (24)	15 (16)
				
Surgical menopause				
Yes	125 (4)	85 (5)	11 (4)	5 (5)
Unknown	10	27	5	1
				
Menopausal symptoms				
Yes	1,469 (57)	1,113 (60)	186 (61)	62 (67)
Unknown	477	29	2	1
				
Socioeconomic status				
Blue collar worker	830 (32)	549 (29)	93 (30)	18 (20)
White collar worker	1,412 (54)	1,081 (58)	175 (57)	60 (67)
Other	337 (14)	248 (13)	39 (13)	12 (13)
Unknown	450	10	1	3
				
Ever use of menopausal hormone therapy				
Any type	1,185 (39)	891 (47)	170 (55)	55 (59)
Unknown	24	5	1	0
				
Estrogen (+/- progestin)	612 (20)	533 (28)	116 (38)	40 (43)
Estradiol	506 (17)	452 (24)	100 (33)	36 (39)
Conjugated estrogens	69 (2)	80 (4)	17 (6)	5 (5)
Other estrogens	19 (0.6)	17 (0.9)	3 (1)	1 (1)
				
Administration of estrogen				
Tablets	526 (17)	465 (25)	103 (34)	39 (42)
Patches	123 (4)	114 (6)	18 (6)	4 (4)
Injections	20 (0.7)	12 (0.6)	1 (0.3)	1 (1)
				
Progestin (+/- estrogen)	462 (15)	420 (22)	95 (31)	38 (41)
Progesterone derived progestin	138 (5)	126 (7)	39 (13)	9 (10)
Testosterone derived progestin	371 (12)	353 (19)	79 (26)	35 (38)
				
Low potency oral estrogen	354 (12)	224 (12)	45 (15)	9 (10)
Local estrogen	374 (12)	260 (14)	33 (11)	12 (13)

**Table 2:** Menopausal hormone therapy and risk of ductal, lobular and tubular cancer

	Controls	Ductal cancer		Lobular cancer		Tubular cancer	
	No.	No.	OR^a ^(95%CI)	No.	OR^a ^(95%CI)	No.	OR^a ^(95%CI)

Regimen							
							
No use	1,707	903	1.0 (Ref)	121	1.0 (Ref)	37	1.0 (Ref)
Medium potency estrogen alone							
Non-exclusive use							
Ever	167	154	1.9 (1.5–2.4)	29	2.5 (1.6–3.9)	12	3.5 (1.8–6.9)
Current	72	67	2.0 (1.4–2.8)	10	2.1 (1.0–4.3)	1	0.7 (0.1–5.6)
Exclusive use^b^							
Ever	76	73	2.0 (1.5–2.9)	12	2.4 (1.3–4.6)	1	0.7 (0.1–5.1)
Duration							
<5 years	50	40	1.6 (1.0–2.5)	6	1.7 (0.7–4.2)	0	-
≥5 years	22	27	2.9 (1.6–5.2)	4	3.1 (1.0–9.5)	1	2.7 (0.3–21.2)
Recency							
Current	31	26	1.6 (1.0–2.8)	6	3.0 (1.2–7.5)	0	-
Past	41	41	2.2 (1.4–3.5)	4	1.5 (0.5–4.2)	1	1.3 (0.2–10.3)
Medium potency estrogen-progestin							
Non-exclusive use							
Ever	350	320	1.6 (1.3–1.9)	79	3.2^c ^(2.3–4.4)	32	4.1^c ^(2.4–7.0)
Current	222	208	1.7 (1.3–2.1)	43	2.8^c ^(1.8–4.2)	16	3.3 (1.7–6.4)
Exclusive use^b^							
Ever	232	215	1.6 (1.3–2.0)	43	2.7^c ^(1.8–4.0)	16	3.2 (1.6–6.1)
Duration							
<5 years	160	137	1.4 (1.1–1.8)	22	1.8 (1.1–3.1)	7	1.9 (0.8–4.7)
≥5 years	62	71	2.3 (1.6–3.3)	21	5.6^c ^(3.2–9.7)	9	6.5^c ^(2.8–14.9)
Recency							
Current	155	171	2.0 (1.5–2.5)	36	3.3^c ^(2.1–5.3)	14	4.2^c ^(2.1–8.8)
Past	67	37	1.0 (0.7–1.5)	7	1.6 (0.7–3.6)	2	1.4 (0.3–6.1)
Sequential medium potency estrogen-progestin^d^							
Non-exclusive use							
Ever	186	199	1.9 (1.5–2.4)	46	3.6^c ^(2.4–5.4)	19	4.5^c ^(2.4–8.3)
Current	88	90	1.8 (1.3–2.4)	12	2.1 (1.1–4.0)	6	2.7 (1.1–7.1)
Exclusive use^b^							
Ever	77	79	1.8 (1.3–2.5)	11	2.1 (1.0–4.2)	5	2.6 (0.9–7.4)
Duration							
<5 years	56	63	1.9 (1.3–2.8)	7	1.7 (0.8–4.1)	4	2.8 (0.9–9.0)
≥5 years	15	16	2.1 (1.0–4.3)	3	3.4 (0.9–12.1)	1	2.7 (0.3–21.7)
Recency							
Current	33	57	2.9 (1.8–4.6)	7	2.9 (1.2–7.1)	4	4.6 (1.4–15.8)
Past	38	22	1.1 (0.6–1.8)	3	1.3 (0.4–4.2)	1	1.2 (0.2–9.0)
Continuous medium potency estrogen-progestin^e^							
Non-exclusive use							
Ever	207	198	1.8 (1.4–2.2)	53	3.7^c ^(2.5–5.4)	20	4.8^c ^(2.6–8.9)
Current	124	92	1.4 (1.0–1.8)	25	2.9^c ^(1.8–4.8)	6	2.3 (0.9–6.0)
Exclusive use^b^							
Ever	114	85	1.4 (1.0–1.9)	22	2.8^c ^(1.6–4.7)	6	2.6 (1.0–6.7)
Duration							
<5 years	86	48	1.0 (0.7–1.4)	13	2.0^c ^(1.0–3.8)	2	1.2 (0.3–5.2)
≥5 years	24	30	2.7 (1.5–4.7)	8	5.9 (2.5–14.0)	4	8.2 (2.4–27.5)
Recency							
Current	90	68	1.4 (1.0–2.0)	18	2.9^c ^(1.6–5.2)	6	3.3 (1.3–8.8)
Past	20	10	0.8 (0.4–1.8)	3	2.0 (0.6–6.9)	0	-
Low potency oral estrogen							
Non-exclusive use							
Ever	314	192	1.2 (1.0–1.5)	42	1.9^c ^(1.3–2.8)	9	1.4 (0.7–3.0)
Current	190	113	1.2 (0.9–1.6)	27	2.0^c ^(1.3–3.2)	4	1.0 (0.3–2.9)
Exclusive use^b^							
Ever	192	113	1.2 (0.9–1.5)	27	2.0^c ^(1.3–3.2)	4	1.0 (0.3–2.8)
Duration							
<5 years	139	87	1.3 (0.9–1.7)	23	2.3^c ^(1.4–3.8)	3	1.0 (0.3–3.4)
≥5 years	50	25	1.1 (0.6–1.8)	4	1.2 (0.4–3.4)	1	1.0 (0.1–7.7)
Recency							
Current	137	72	1.1 (0.8–1.5)	13	1.4 (0.7–2.5)	2	0.7 (0.2–3.1)
Past	52	40	1.6 (1.0–2.4)	14	3.8^c ^(2.0–7.1)	2	1.6 (0.4–6.9)
Local estrogen							
Non-exclusive use							
Ever	349	226	1.3 (1.0–1.5)	31	1.3 (0.8–1.9)	10	1.3 (0.7–2.8)
Current	229	130	1.1 (0.9–1.4)	13	0.8 (0.4–1.5)	7	1.5 (0.7–3.5)
Exclusive use^b^							
Ever	236	133	1.1 (0.9–1.4)	14	0.8 (0.5–1.5)	7	1.5 (0.6–3.4)
Duration							
<5 years	165	90	1.0 (0.8–1.3)	11	0.9 (0.5–1.8)	5	1.5 (0.6–4.0)
≥5 years	62	39	1.3 (0.8–1.9)	2	0.5 (0.1–1.9)	2	1.6 (0.4–7.0)
Recency							
Current	168	94	1.1 (0.8–1.4)	10	0.9 (0.4–1.7)	6	1.8 (0.8–4.5)
Past	59	35	1.1 (0.7–1.7)	3	0.7 (0.2–2.2)	1	0.7 (0.1–5.6)

Women with unknown age at menopause were excluded. Recency: Current = last use within 6 months before reference date (see methods for definition or reference date); Past = last use > 6 months before reference date. ^a^Adjusted for age, age at first birth, age at menopause and recent body mass index (one year before data collection). ^b^Users of more than one kind of menopausal hormone therapy excluded. ^c^Statistically significant difference compared with the estimate for ductal cancer. ^d^Sequential estrogen-progestin: progestin included <16 days per 28 days. ^e^Continuous estrogen-progestin: progestin included > 19 days per 28 days. CI, confidence interval; OR, odds ratio; Ref, reference category.

**Table 3:** Various breast cancer risk factors and risk of ductal, lobular and tubular breast cancer

	Controls	Ductal cancer		Lobular cancer		Tubular cancer	
	No.	No.	OR^a ^(95%CI)		OR^a ^(95%CI)	No.	OR^a ^(95%CI)

Factor							
Parous							
Nulliparous	337	292	1.0 (Ref)	40	1.0 (Ref)	10	1.0 (Ref)
Parous	2,643	1,561	0.7 (0.6–0.8)	258	0.8 (0.6–1.2)	81	1.0 (0.5–2.0)
No of births^b^							
1	558	416	1.0 (Ref)	47	1.0 (Ref)	21	1.0 (Ref)
2	1073	690	0.9 (0.7–1.0)	126	1.5^c ^(1.0–2.1)	36	0.9 (0.5–1.6)
3	630	318	0.7 (0.6–0.9)	58	1.3^c ^(0.8–1.9)	19	0.9 (0.5–1.8)
≥4	382	137	0.6 (0.4–0.7)	27	1.1^c ^(0.7–1.9)	5	0.5 (0.2–1.3)
Age at first birth (years)^b^							
<20	305	143	0.9 (0.7–1.1)	20	0.7 (0.4–1.1)	9	1.3 (0.6–2.9)
20–24	1,125	600	1.0 (Ref)	106	1.0 (Ref)	25	1.0 (Ref)
25–29	836	526	1.1 (1.0–1.3)	79	1.0 (0.7–1.4)	29	1.5 (0.9–2.6)
≥30	377	292	1.3 (1.1–1.6)	53	1.6 (1.1–2.3)	18	1.9 (1.0–3.7)
Age at menarche (years)^d^							
<12	164	134	1.3 (1.0–1.7)	23	1.3 (0.8–2.1)	4	0.7 (0.2–1.9)
12–13	1,155	702	1.0 (Ref)	117	1.0 (Ref)	38	1.0 (Ref)
14–15	1,161	723	1.1 (0.9–1.2)	109	1.0 (0.7–1.3)	43	1.2 (0.7–1.8)
≥16	236	113	0.8 (0.6–1.0)	23	1.0 (0.6–1.5)	3	0.4 (0.1–1.2)
Age at menopause (years)^e^							
<45	328	144	0.7 (0.5–0.8)	22	0.7 (0.4–1.1)	4	0.4 (0.1–1.1)
45–49	658	368	0.8 (0.7–1.0)	68	1.0 (0.8–1.4)	24	1.2 (0.7–2.0)
50–54	1,236	805	1.0 (Ref)	121	1.0 (Ref)	37	1.0 (Ref)
≥55	268	174	1.0 (0.8–1.3)	27	1.1 (0.7–1.7)	3	0.4 (0.1–1.2)
Height (cm)							
<160	652	361	1.0 (Ref)	46	1.0 (Ref)	16	1.0 (Ref)
160–164	1,012	593	1.0 (0.9–1.2)	110	1.5^c ^(1.1–2.2)	27	1.0 (0.5–1.8)
165–169	880	555	1.1 (0.9–1.3)	86	1.3 (0.9–1.9)	27	1.1 (0.6–2.1)
≥170	436	344	1.3 (1.1–1.6)	57	1.7 (1.1–2.6)	21	1.7 (0.9–3.3)
Recent BMI (kg/m^2^)^f^							
<22.2	430	165	1.0 (Ref)	26	1.0 (Ref)	9	1.0 (Ref)
22.2–<24.2	423	214	1.3 (1.0–1.7)	25	1.0 (0.6–1.7)	10	1.1 (0.4–2.8)
24.2–<26.0	427	217	1.3 (1.0–1.7)	21	0.8 (0.5–1.5)	9	1.0 (0.4–2.5)
26.0–<28.5	420	251	1.6 (1.2–2.0)	39	1.6 (0.9–2.6)	9	1.0 (0.4–2.6)
≥28..5	430	301	1.9 (1.5–2.3)	41	1.6 (1.0–2.6)	9	1.0 (0.4–2.6)
Adult weight gain (kg)^d,f,g^							
<0	159	64	0.8 (0.6–1.1)	11	0.9 (0.5–1.9)	4	0.8 (0.3–2.4)
0–9.5	459	242	1.0 (Ref)	34	1.0 (Ref)	15	1.0 (Ref)
10–19.5	534	349	1.2 (1.0–1.5)	44	1.1 (0.7–1.8)	13	0.7 (0.3–1.6)
20–29.5	226	171	1.5 (1.1–1.9)	21	1.2 (0.7–2.2)	6	0.8 (0.3–2.2)
≥30	99	75	1.5 (1.1–2.1)	13	1.8 (0.9–3.5)	6	2.0 (0.7–5.3)
Mother or sister with breast cancer^d^							
No	2,294	1,530	1.0 (Ref)	237	1.0 (Ref)	73	1.0 (Ref)
Yes	233	278	1.8 (1.5–2.2)	57	2.4 (1.8–3.4)	15	2.2 (1.2–3.9)
Operated due to benign breast disease^d^							
No	2,315	1,590	1.0 (Ref)	245	1.0 (Ref)	82	1.0 (Ref)
Yes	242	253	1.5 (1.2–1.8)	53	1.9 (1.4–2.6)	9	0.9 (0.4–1.8)
Oral contraceptive use^d^							
No use	2,030	1,237	1.0 (Ref)	198	1.0 (Ref)	55	1.0 (Ref)
Duration							
<5 years	470	302	0.9 (0.7–1.0)	40	0.6 (0.4–0.9)	23	1.3 (0.7–2.2)
≥5 years	331	215	0.9 (0.7–1.1)	42	0.9 (0.6–1.4)	13	1.0 (0.5–1.9)
Recent alcohol consumption (g/day)^d^							
No alcohol	1,108	762	1.0 (Ref)	111	1.0 (Ref)	28	1.0 (Ref)
<5	905	674	1.0 (0.9–1.2)	101	1.0 (0.8–1.4)	34	1.2 (0.7–2.1)
≥5–10	210	171	1.1 (0.9–1.4)	32	1.3 (0.9–2.1)	12	1.8 (0.9–3.6)
>10	100	72	1.0 (0.7–1.4)	19	1.6 (0.9–2.8)	10	3.1^c ^(1.4–6.8)
Recent smoking^d^							
No	2,212	1,442	1.0 (Ref)	229	1.0 (Ref)	63	1.0 (Ref)
Yes	696	408	1.2 (1.0–1.4)	68	1.1 (0.8–1.5)	28	0.8^c ^(0.5–1.2)

Recent = one year before data collection. ^a^Adjusted for age, and where applicable also for estrogen-progestin use (ever/never). ^b^Number of births and age at first birth were adjusted for each other. ^c^Statistically significant difference compared with the estimate for ductal cancer. ^d^Some cases and controls excluded due to missing information. ^e^Women with unknown age at menopause and women using menopausal hormone therapy apart from local estrogen before menopause excluded. ^f^Women ever menopausal hormone therapy apart from local estrogen excluded. ^g^Weight gain from 18 years of age to one year before data collection. BMI, body mass index; CI, confidence interval; OR, odds ratio; Ref, reference category.

**Table 4:** Medium potency estrogen-progestin therapy and risk of ductal and lobular breast cancer stratified by body mass index

Recent BMI	Control group		Ductal cancer				Lobular cancer			
	Never use	Ever use	Never use		Ever use		Never use		Ever use	

	No.	No.	No.	OR^a ^(95%CI)	No.	OR^a ^(95%CI)	No.	OR^a ^(95%CI)	No.	OR^a ^(95%CI)

BMI <22	298	47	126	1.0 (Ref)	49	2.0 (1.2–3.3)	19	1.0 (Ref)	12	3.4 (1.4–8.3)
BMI 22–27	895	132	420	1.1 (0.9–1.4)	114	1.6 (1.1–2.3)	46	0.8 (0.4–1.4)	21	2.2 (1.0–4.6)
BMI >27	514	53	357	1.8 (1.4–2.3)	52	1.9 (1.2–3.1)	56	1.9 (1.1–3.3)	10	2.2 (0.9–5.6)

Exclusive use of medium potency estrogen-progestin therapy, users of more than one kind of menopausal hormone therapy were excluded. Women with unknown age at menopause were excluded. Recent = one year before data collection. ^a^Adjusted for age, age at first birth and age at menopause. BMI, body mass index; CI, confidence interval; OR, odds ratio; Ref, reference category.
